# Association of Conditional Cash Transfers With Maternal Mortality Using the 100 Million Brazilian Cohort

**DOI:** 10.1001/jamanetworkopen.2023.0070

**Published:** 2023-02-23

**Authors:** Flávia Jôse O. Alves, Dandara Ramos, Enny S. Paixão, Ila R. Falcão, Rita de Cássia Ribeiro-Silva, Rosemeire Fiaccone, Davide Rasella, Camila Teixeira, Daiane Borges Machado, Aline Rocha, Marcia F. de Almeida, Emanuelle F. Goes, Laura C. Rodrigues, Maria Yury Ichihara, Estela M. L. Aquino, Maurício L. Barreto

**Affiliations:** 1 Institute of Collective Health, Federal University of Bahia, Salvador, Brazil; 2 Center for Data and Knowledge Integration for Health (CIDACS), Oswaldo Cruz Foundation, Salvador, Brazil; 3 Iyaleta Research Association, Salvador, Brazil; 4 Infectious Disease Department, Faculty of Epidemiology and Population Health, London School of Hygiene and Tropical Medicine, London, United Kingdom; 5 School of Nutrition, Federal University of Bahia, Salvador, Brazil; 6 Department of Statistics, Federal University of Bahia, Salvador, Brazil; 7 ISGlobal, Hospital Clínic, Universitat de Barcelona, Barcelona, Spain; 8 Department of Global Health and Social Medicine, Harvard Medical School, Boston, Massachusetts; 9 School of Public Health, University of São Paulo, São Paulo, Brazil

## Abstract

**Importance:**

Conditional cash transfers (CCTs) have been consistently associated with improvements to the determinants of maternal health, but there have been insufficient investigations regarding their effects on maternal mortality.

**Objective:**

To evaluate the association between being a *Bolsa Família* program (BFP) beneficiary and maternal mortality and to examine how this association differs by duration of BFP receipt, maternal race, living in rural or urban areas, the Municipal Human Development Index (MHDI), and municipal primary health care coverage.

**Design, Setting, and Participants:**

This cross-sectional analysis was nested within the 100 Million Brazilian Cohort. Girls and women aged 10 to 49 years (hereinafter referred to as women) who had at least 1 live birth were included, using data from Brazilian national health databases linked to the 100 Million Brazilian Cohort (January 1, 2004, to December 31, 2015). Propensity score kernel weighting was applied to control for sociodemographic and economic confounders in the association between BFP receipt and maternal mortality, overall and stratified by different subgroups (race, urban or rural area, and MHDI), and duration of BFP receipt. Data were analyzed from July 12, 2019, to December 31, 2022.

**Main Outcome(s) and Measures:**

Maternal death.

**Results:**

A total of 6 677 273 women aged 10 to 49 years were included in the analysis, 4056 of whom had died from pregnancy-related causes. The risk of maternal death was 18% lower in women who received BFP (weighted odds ratio [OR], 0.82 [95% CI, 0.71-0.93]). A longer duration receiving BFP was associated with an increased reduction in maternal mortality (OR for 1-4 years, 0.85 [95% CI, 0.75-0.97]; OR for 5-8 years, 0.70 [95% CI, 0.60-0.82]; OR for ≥9 years, 0.69 [95% CI, 0.53-0.88]). Receiving BFP was also associated with substantial increases in the number of prenatal appointments and interbirth intervals. The reduction was more pronounced in the most vulnerable groups.

**Conclusions and Relevance:**

This cross-sectional analysis nested within the 100 Million Brazilian Cohort found an association between BFP receipt and maternal mortality. This association was of greater magnitude in women with longer exposure to BFP and in the most vulnerable groups. These findings reinforce evidence that programs such as BFP, which have already proven effective in poverty reduction, have great potential to improve maternal survival.

## Key Points

**Question:**

What is the effect of the world’s largest conditional cash transfer program, *Bolsa Família* (BFP), on maternal mortality?

**Findings:**

This analysis nested within the 100 Million Brazilian Cohort, including more than 6 million women, found an association of BFP with reducing maternal mortality. A longer duration of receiving BFP was associated with a higher reduction in maternal mortality, and the poorest women benefited most from BFP.

**Meaning:**

These findings suggest that programs such as BFP have a potential to reduce maternal mortality and health inequalities.

## Teaser

This cohort study uses national Brazilian linked data from health and social administrative databases to investigate the association between the *Bolsa Família* conditional cash transfer program and maternal mortality among women and girls of child-bearing age.

## Introduction

Many women, especially those from low and middle-income countries, experience pregnancy-related complications, with increased risk of death.^1^ Maternal mortality remains a looming problem in contexts of inequality, as in the US, which maintains high rates (23.8 deaths per 100 000 live births in 2020), with those for non-Hispanic Black women almost 3 times higher (55.3 deaths per 100 000 live births) than for non-Hispanic White women (19.1 deaths per 100 000 live births).^2^ In Brazil, the maternal mortality rate remains high (57.0 deaths per 100 000 live births in 2019), with a modest improvement in the last decade.^3^

Given that most maternal and child deaths are poverty-related,^4,5^ conditional cash transfers (CCTs) have been proposed as a promising strategy to promote maternal and child survival.^6^ While some CCTs aim to alleviate poverty and increase human capital through transfers that are conditioned on school attendance and the use of maternal and child health services (child welfare, maternal and child vaccination, and prenatal attendance), other CCT transfers, such as India’s Janani Suraksha Yojana (JSY) and Nepal’s Safe Delivery Incentive Program, transfer cash only for use of specific services, such as health facility–based delivery.^4^ Despite these differences, studies have shown consistent associations between CCTs and reduction of poverty and improved access to health care services,^4,5^ which are considered strategic actions to decrease maternal mortality.^4,5^ However, most of these studies are generally performed with small samples. There is still little knowledge of whether CCT programs have led to improvements in maternal health outcomes, such as maternal mortality, and whether their effects are generalizable across different settings. A study of the Mexican CCT *Oportunidades* found an 11% reduction in maternal mortality,^7^ and another study of the Indian CCT JSY showed no significant association.^8^ A recent Brazilian study using ecological data from municipalities over a 11-year period^9^ demonstrated the association of CCTs with reductions in maternal mortality. The association increased with the increment in coverage levels and years of implementation.^9^

The mechanisms through which the program can affect maternal mortality were proposed and described in the research protocol^10^ and a previous study.^9^ Like other CCTs, BFP can affect maternal health both through receipt of the benefit and conditionalities. Income transfers can have a more immediate effect with the allocation of money for the purchase of food and other necessities and the use of health services. On the other hand, these health requirements impact the use of services during pregnancy and puerperium.^4,5^ In this study, drawing from a large, longitudinal populational-based cohort study using national Brazilian linked data from health and social administrative databases (the 100 Million Brazilian Cohort), we investigated the association between BFP and maternal mortality. We hypothesize that BFP is associated with decreased maternal mortality and that this association is more prominent with the increased duration of the benefit.

## Methods

This cohort study was approved by the research ethics committee of the Federal University of Bahia, Salvador, Brazil. Data were stored on secure servers on the Center for Data and Knowledge Integration for Health (CIDACS) Big Data Integrated Platform.^11^ Since no personally identifiable information was included in the data set used for analysis, informed consent was waived. The study followed the Strengthening the Reporting of Observational Studies in Epidemiology (_STROBE_) reporting guideline.

All of the methods and analyses were described in the previously published research protocol.^10^ We conducted a cross-sectional analysis nested within the 100 Million Brazilian Cohort, a retrospective dynamic cohort resulting from linkage of national administrative, social, and health data sets.^12^ The cohort baseline includes records from over 114 million individuals in the Unified Registry for Social programs (*Cadastro Único*)*,* an administrative system with detailed information on the poor and extremely poor in Brazil (families with a monthly income ≤3 minimum wages [approximately US $750]) (eMethods in Supplement 1). Detailed information on the 100 Million Brazilian Cohort is available on its profile.^13^

Our study population consisted of girls and women aged 10 to 49 years (hereinafter referred to as women), registered on the cohort baseline, who had at least 1 live birth in their last pregnancy between January 1, 2004, and December 31, 2015. To reduce selection biases and to produce the best comparative group for women who had died from maternal causes, we only included the woman’s most recent delivery in the cohort and not all of the deliveries during her lifetime.

### Linkage Process

We identified all of the women who had at least 1 live birth by connecting the live birth information system (*Sistema de Informações sobre Nascidos Vivos* [SINASC]) with the 100 Brazilian Million Cohort baseline. To identify maternal death records and causes of death in the cohort, we then linked the matched pairs of the first stage (SINASC and 100 Million Brazilian Cohort baseline) to records of pregnancy-related causes within the mortality information system (*Sistema de Informações sobre Mortalidade*). These linkages were conducted by similarity scores, using the CIDACS record linkage tool, an innovative algorithm developed to connect large-scale administrative data sets,^14,15,16^ based on their similarity across several identifiers. In our case, the linkage attributes were the woman’s name and age at the time of delivery, date of birth, and the municipality of residence at the time of delivery. Analysis of the linkage accuracy included manual verification of a randomly selected sample of records, assessing the receiver operating characteristics curve of sensitivity, and specificity indexes^14^ (eFigure 3 in Supplement 1). In this validation process, we obtained a mean sensitivity and specificity of over 92% (eFigure 3 and eTable 18 in Supplement 1).

### Exposure

*Bolsa Família* is a CCT program that aims to reduce poverty by providing a monthly benefit and to break the intergenerational poverty of its beneficiaries through health and education conditionalities.^17^ Women and their families can receive basic and/or variable benefits, with values according to their level of income, considering groups of extreme poverty and poverty. Families who receive BFP are required to fulfill educational (a minimum of 85% school attendance) and health (health care appointments and a vaccination schedule) conditionalities, with a variable benefit intended for pregnant and lactating women.^18^ Since BFP was implemented in 2004, women registered on *Cadastro Único* before 2004 were considered the first to benefit from social programs on January 1, 2004. We deemed those exposed to BFP as all women with records of live births who started receiving BFP before or during pregnancy and did not stop receiving the benefit until childbirth or before death. Women who had not received the benefit at any time or until delivery were considered not exposed. Women who stopped receiving the benefit at some point before childbirth were excluded from the analysis.

### Outcome

Our main study outcome was maternal death, according to code XV, O00 to O99 from the *International Statistical Classification of Diseases and Related Health Problems, Tenth Revision* (*ICD-10*). Conditions classified in other chapters of *ICD-10* were also included (A34, F53, M83.0, B20-B24, D39.2, and E23.0), provided that these deaths occurred within 42 days following delivery.^19^ We were not able to include deaths that occurred before delivery, due to linkage with SINASC.

### Statistical Analysis

All analyses were performed from July 12, 2019, to December 31, 2022, using Stata, version 15.0 (StataCorp LLC). Two-sided *P* < .05 indicated statistical significance. In line with the published research protocol^10^ and previous quasi-experimental studies using the *Cadastro Único* data set and the 100 Million Brazilian Cohort,^20,21,22^ the association between BFP and maternal mortality was estimated based on the propensity score–based method. First, we used logistic regression to estimate the conditional probability of receiving treatment (being a BFP beneficiary or not), given the set of observable characteristics using the propensity score (eFigures 1 and 2 and eTable 2 in Supplement 1).^23^ Since whether a family receives the benefit or not is determined by per capita income and a set of family socioeconomic characteristics, the following covariates were considered to estimate the propensity score: self-reported race (5 categories available in *Cadastro Único*, according to the Brazilian Institute of Geography and Statistics classification [definitions provided hereinafter]),^19^ level of education, age, parity (number of childbirths in the cohort), location of the household (urban or rural residency), Brazilian region, household density, type of water supply, waste disposal system (sewer system), and garbage disposal. *Cadastro Único* was created in 2001, and the registry has been improved and expanded over the years.^8^ With creation of BFP (2004), there was a greater incentive to attract families in situations of poverty and extreme poverty to *Cadastro Único* and, in 2006, BFP achieved full expansion.^24^ To solve any problems that may arise with this variation in the registry over time, we also incorporated the year of registration on *Cadastro Único* for propensity score estimation.

As a second step, we performed a kernel-matching approach, which uses weights based on the propensity score to select observations of nonbeneficiaries who are more similar to the set of beneficiaries.^25^ In this study, we estimate the standard error by the bootstrap method, which was based on a resampling estimate with substitution of the original sample.^26,27^ As a third step, we estimated the mean treatment effect on the treated, after applying the matching weights (eTable 3 in Supplement 1) estimated through the kernel process. As a fourth and final step, we adjusted weighted logistic regression models with 95% CIs between BFP receipt and maternal death. To control for risk factors of maternal mortality (eMethods in Supplement 1, section 3.5),^25^ we also adjusted multivariate models for other covariates not used to estimate the propensity score: prenatal care (none, 1-3, 4-6, or ≥7 appointments), type of delivery (vaginal or cesarean), gestational period (<22, 22-27, 28-31, 32-36, 37-41, and ≥42 weeks), and multiple pregnancy (yes or no). We compared the differences in the distribution of propensity score covariates between beneficiaries and nonbeneficiaries, using proportions to assess the covariate balance before and after kernel weighting.

#### Duration of Receipt

To evaluate exposure to BFP, according to benefit duration, we estimated the kernel-matching and weighted logistic regressions, comparing women exposed to different ranges of years between BFP receipt until delivery (1-4, 5-8, or ≥9 years) with those not exposed to the program. In addition, since the exposure time may differ according to the woman’s age, we calculated an indicator considering the years of BFP exposure, divided by the woman’s age on delivery, multiplied by 100, resulting in a proxy of the percentage of the lives of women exposed to BFP until delivery (mean [SD], 30.44% [16.06%]), classified as less than 30%, 30% to 69%, and 70% or greater.

#### Subgroup Analysis

To evaluate possible effect modifications of the association between BFP exposure and maternal mortality, we conducted an analysis stratified by subgroups of sociodemographic indicators, such as area of residence (rural and urban), Municipal Human Development Index (MHDI; high or very high, medium, low, and very low), and race (Black, *Pardo* [which translates from Portuguese as “brown” or “mixed” and is used to denote individuals with predominantly Black and also mixed ancestry, including European, African, and Indigenous backgrounds^28,29^], and White). Racial categories were based on Brazilian Institute of Geography and Statistics terminology, which reflects Brazilian concepts of race and gives mothers the option of categorizing themselves as Asian, Black, *Pardo*, Indigenous, or White. We estimated the propensity score for each subgroup of these sociodemographic indicators, with the same variables in the previous steps, and conducted kernel-weighted logistic analysis with models separately within each subgroup. We also investigated the association between BFP and maternal mortality by the mean municipal Family Health Program (FHP) coverage, as stratified in a previous study (≤30%, 30%-70%, or >70%),^30^ and conducted multivariate logistic regression adjusted for the same propensity score variables, exploring the association of receiving BFP with prenatal care (<4 or ≥4 appointments) and the interbirth interval (<24 or ≥24 months or 1 live birth in the cohort). The maternal mortality rate was measured by the number of maternal deaths over the number of women who had at least 1 live birth, to provide a closer approximation of risk in the cohort.^31^

#### Sensitivity Analysis

We performed the following analyses to assess the robustness of the results. First, to investigate possible biases in the kernel-weighting procedure, we estimated the association of BFP using the inverse probability of treatment weighting, estimating the weights for BFP beneficiaries (weight = 1) and nonbeneficiaries using Weight = Propensity Score/[1 − Propensity Score].^32^ We also performed an analysis using conventional, multivariate regression models adjusted for confounders (eTables 4 and 5 in Supplement 1). Second, to verify whether different definitions of BFP exposure would affect the results, we tested other classifications for nonexposure and exposure, considering the period of pregnancy covered by exposure (eTables 6 and 7 in Supplement 1). Third, to test whether the results considering different ranges of years between BFP receipt until delivery (1-4, 5-8, or ≥9 years) were consistent for each year, we also estimated kernel-matching and weighted logistic regressions for each year of the duration of BFP receipt in the study period (eTables 8-10 in Supplement 1). To assess the robustness of the variables used for matching, we also performed analyses by removing variables from propensity score housing conditions (eTables 15-18 in Supplement 1).

## Results

**Figure 1.**
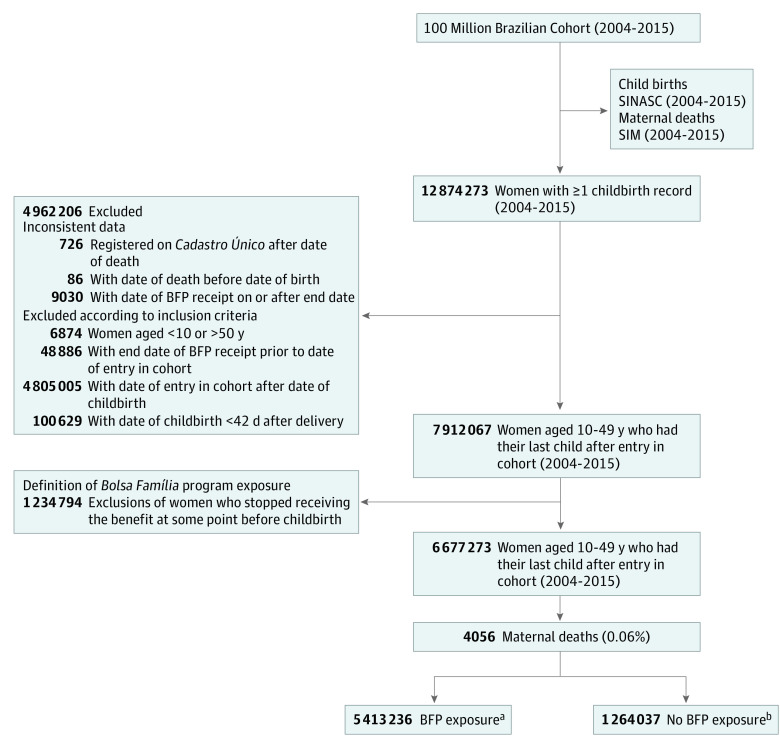
Flowchart of Study Population Definition SIM indicates mortality information system (*Sistema de Informações sobre Mortalidade*); SINASC, live birth information system (*Sistema de Informações sobre Nascidos Vivos*). ^a^Includes women who started receiving *Bolsa Família* program (BFP) benefit before or during pregnancy and did not stop receiving the benefit until childbirth, or before death. ^b^Includes women who did not receive the benefit at any time or until childbirth.

Following definition of BFP exposure, we included 6 677 273 women aged 10 to 49 years who had at least 1 live birth, and 4056 of these had died of pregnancy-related causes. A total of 5 413 236 (81.07%) were BFP beneficiaries and 1 264 037 (18.93%) were nonbeneficiaries (Figure 1). The characteristics of the population excluded from the analysis on account of the BFP exposure definition (women who stopped receiving the benefit at some point before childbirth) are described in eTable 1 in Supplement 1.

At baseline, BFP beneficiaries were more likely to be younger, Black or *Pardo*, have a lower level of education, reside in rural areas, and have worse housing conditions (Table 1). Compared with baseline characteristics, the 2 groups became more similar after kernel weighting (Table 1). Lower maternal mortality rates were observed among BFP beneficiaries after kernel-weighting balancing (60.63 [95% CI, 58.46-62.89] vs 71.59 [95% CI, 62.87-81.53] deaths per 100 000 women who had live births). In the model adjusted for the number of prenatal appointments, type of delivery, gestational period, and multiple pregnancies, BFP receipt was associated with an 18% decreased maternal mortality (weighted odds ratio [OR], 0.82 [95% CI, 0.71-0.93]) in the kernel-weighting regression (Table 2).

**Table 1.** Description of BFP Nonbeneficiaries and Beneficiaries Before and After Kernel Weighting, 2004 to 2015 (N = 6 677 273)

Variable	Before kernel weighting, %			After kernel weighting, %		
	Non-BFP (n = 1 264 037)	BFP (n = 5 413 236)	Diff (BFP-Non-BFP	Non-BFP (n = 1 017 154)	BFP (n = 4 731 624)	Diff (BFP-Non-BFP)
Race						
Asian	0.47	0.35	−0.12	0.31	0.34	0.03
Black	7.36	8.78	1.42	8.56	8.74	0.18
Indigenous	0.29	1.03	0.74	0.70	0.80	0.10
* Pardo*	55.69	63.69	8.00	64.04	63.57	−0.47
White	36.19	26.15	−10.04	26.39	26.49	0.10
Missing data^a^	9.35	5.76	NA	NA	NA	NA
Educational attainment						
High school or college (≥8 y)	69.64	58.88	−10.76	57.82	58.78	0.96
Elementary or middle school (4-7 y)	24.21	32.86	8.65	33.68	33.02	−0.66
Elementary school or illiterate (<3 y)	6.15	8.26	2.11	8.50	8.20	−0.30
Missing data^a^	1.98	2.26	NA	NA	NA	AN
Age group, y						
10-19	14.09	23.24	9.15	23.47	23.66	0.19
20-34	75.89	69.32	−6.57	69.45	68.88	−0.57
≥35	10.02	7.44	−2.58	7.08	7.46	0.38
Missing data^a^	0	0	NA	NA	NA	NA
No. of children in cohort						
1	39.74	44.35	4.61	45.46	44.21	−1.25
2-3	57.80	49.66	−8.14	48.90	49.87	0.97
>3	2.46	5.99	3.53	5.64	5.92	0.28
Missing data^a^	0	0	NA	NA	NA	NA
Household density						
≤2 Persons per room	79.30	53.22	−26.08	53.21	53.33	0.12
>2 Persons per room	20.70	46.78	26.08	46.79	46.67	−0.12
Missing data^a^	11.63	5.10	NA	NA	NA	NA
Water supply						
Public network	77.51	65.42	−12.09	65.64	65.98	0.34
Well, natural source, or other	22.49	34.58	12.09	34.36	34.01	−0.35
Missing data^a^	7.53	2.79	NA	NA	NA	NA
Waste disposal system						
Public network	77.51	38.72	−38.79	38.28	38.81	0.53
Septic tank, ditch, or other	22.49	61.28	38.79	61.72	61.19	−0.53
Missing data^a^	8.88	3.51	NA	NA	NA	NA
Garbage disposal						
Public collection system	51.21	68.78	17.57	69.35	69.77	0.42
Burned, buried, or other	49.79	31.22	−18.57	30.64	30.22	−0.42
Missing data^a^	7.53	2.79	NA	NA	NA	NA
Geographical region						
South	14.19	8.55	−5.64	8.43	8.71	0.28
North	9.78	13.21	3.43	12.99	12.74	−0.25
Northeast	30.69	42.87	12.18	42.80	42.86	0.06
Southeast	35.10	29.29	−5.81	29.66	29.68	0.02
Center-West	10.24	6.08	−4.16	6.12	6.01	−0.11
Missing data^a^	0	0	NA	NA	NA	NA
Location of household						
Urban	81.56	71.74	−9.82	72.15	72.40	0.25
Rural	18.44	28.26	9.82 ^b^	27.85	27.60	−0.25^b^
Missing data^a^	5.56	2.06	NA	NA	NA	NA
Year						
2004	7.85	10.26	2.41	10.39	10.55	0.16
2005	7.12	7.92	0.80 ^b^	7.53	7.85	0.32^b^
2006	26.84	45.62	18.78	46.44	46.69	0.25
2007	11.18	13.71	2.53	14.64	14.44	−0.2
2008	5.37	5.53	0.16	6.06	5.84	−0.22
2009	4.33	4.85	0.52	5.22	5.09	−0.13
2010	6.12	4.36	−1.76	3.59	3.50	−0.09
2011	5.18	2.51	−2.67	1.56	1.58	0.02
2012	10.41	2.79	−7.62	2.61	2.54	−0.07
2013	7.44	1.61	−5.83	1.17	1.16	−0.01
2014	6.09	0.72	−5.37	0.67	0.65	−0.02
2015	2.07	0.12	−1.95	0.12	0.11	−0.01
Missing data^a^	0	0	NA	NA	NA	NA

Abbreviations: BFP, *Bolsa Família* program; NA, not applicable.

^a^ Percentage was not included when calculating the categories.

^b^ *P* < .005. For all others in these columns, *P* < .001.

**Table 2.** Kernel-Weighted Regression for Associations Between BFP Receipt and Maternal Death^a^

	Kernel-weighted rate, % (95% CI)		OR (95% CI)	
	Non-BFP^b^	BFP^b^	Nonadjusted	Adjusted^c^
Rate or OR	71.59 (62.87-81.53)	60.63 (58.46-62.89)	0.84 (0.73-0.96)	0.82 (0.71-0.93)
No. of women	1 017 154	4 731 624	5 748 917	5 542 230

Abbreviations: BFP, *Bolsa Família* program; OR, odds ratio.

^a^ Data are from the 100 Million Brazilian Cohort, 2004 to 2015.

^b^ Non-BFP and BFP correspond to kernel-weighted maternal rates per 100 000 parturients of the live birth information system (*Sistema de Informações sobre Nascidos Vivos*) to nonbeneficiaries and beneficiaries, respectively.

^c^ Adjusted by prenatal care, gestational age, type of delivery, and multiple pregnancy.

**Figure 2.**
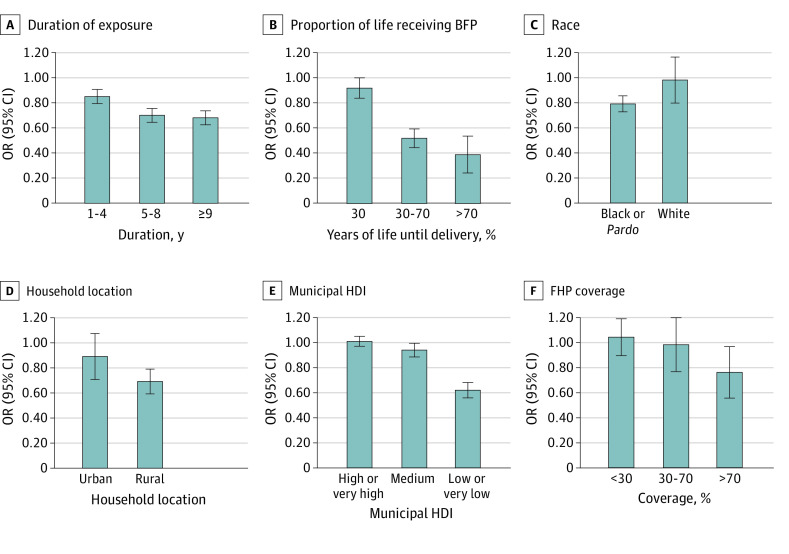
Receipt of *Bolsa Família* program (BFP) Benefit, Sociodemographic Vulnerability Markers, and Duration of BFP Receipt Kernel-weighted logistic regression was used for associations between BFP receipt and maternal death by time of exposure and BFP participation by years of life until delivery (ie, years of receipt divided by years of life until delivery). Stratified analysis by sociodemographic vulnerability markers included race, location of household, Municipal Human Development Index (HDI), and Family Health Program (FHP) coverage. All the analytical steps (propensity score estimation, kernel-matching and weighted logistic regressions) were conducted separately for each category of this subgroup. *Pardo* translates from Portuguese as “brown” or “mixed” and is used to denote individuals with predominantly Black and also mixed ancestry, including European, African, and Indigenous backgrounds. OR indicates odds ratio.

Increased duration of BFP exposure was associated with a reduction in maternal mortality (weighted OR for 1-4 years, 0.85 [95% CI, 0.75-0.97]; weighted OR for 5-8 years, 0.70 [95% CI, 0.60-0.82]; and weighted OR for ≥9 years, 0.69 [95% CI, 0.53-0.88]). The higher the percentage of the woman’s lifetime exposed to BFP on delivery, the greater the chances were of reducing maternal death (weighted OR for <30%, 0.92 [95% CI, 0.77-1.09]; weighted OR for 30%-70%, 0.52 [95% CI, 0.40-0.69]; and weighted OR for >70%, 0.39 [95% CI, 0.18-0.82]). A higher protective effect of BFP was observed among Black or *Pardo* women (weighted OR, 0.79 [95% CI, 0.69-0.93]), women living in rural areas (weighted OR, 0.69 [95% CI, 0.53-0.92]), and women who lived in less developed municipalities (weighted OR for low or very low MHDI, 0.62 [95% CI, 0.47-0.84]), although the 95% CIs overlapped (Figure 2 and eTables 8-10 and 11-13 in Supplement 1). Receiving BFP was associated with the reduction of maternal mortality among women living in municipalities with high FHP coverage (weighted OR for ≥70%, 0.76 [95% CI, 0.57-0.97]; weighted OR for 30%-70%, 0.98 [95% CI, 0.78-1.22]; weighted OR for <30%, 1.04 [95% CI, 0.78-1.39]) (Figure 2 and eTable 14 in Supplement 1).

The BFP beneficiaries were more likely to attend 4 or more prenatal appointments (adjusted OR, 1.43 [95% CI, 1.42-1.44]). They also had a longer interbirth interval than nonbeneficiaries (adjusted OR, 1.39 [95% CI, 1.39-1.40]) (Table 3).

**Table 3.** Logistic Regression for Associations Between BFP Participation, Prenatal Care Appointments, and Interpregnancy Intervals^a^

Variable	Non-BFP	BFP	Adjusted OR (95% CI)^b^
Prenatal care appointments			
<4	14 087 (1.25)	435 718 (8.12)	1 [Reference]
≥4	1 112 322 (98.75)	4 930 276 (91.88)	1.43 (1.42-1.44)
No. of women	1 126 409	5 365 994	6 619 173
Interpregnancy interval, mo			
<24	509 786 (40.33)	1 769 593 (32.98)	1 [Reference]
≥24	754 251 (59.67)	3 596 401 (67.02)	1.39 (1.39-1.40)
No. of women	1 264 037	5 365 994	6 677 293

Abbreviations: BFP, *Bolsa Família* program; OR, odds ratio.

^a^ Data are from the 100 Million Brazilian Cohort, 2004 to 2015.

^b^ Multivariate logistic regression adjusted by propensity score variables.

### Sensitivity Analysis

We obtained similar point estimates for the association between receiving BFP and decreased maternal mortality in all of the sensitivity analyses performed. Details are found in eTables 4 to 10 and 15 to 17 in Supplement 1.

## Discussion

Our findings in this cohort study suggest that BFP is associated with a reduction in maternal mortality among the poorest Brazilian women. This association remained robust after adjustment for health care, multiple pregnancies, and type of delivery, achieving an 18% decrease in the chance of maternal mortality among BFP beneficiaries. Increased exposure to BFP increased the magnitude of the association. Receipt of BFP was also associated with prenatal appointments and increased interbirth interval. There was also an association among the most vulnerable groups, such as Black or *Pardo* women and municipalities with the lowest MHDI.

Several studies have shown that CCT programs have had a positive association with the social determinants of maternal health, such as nutritional status, immunization coverage, the promotion of healthy behaviors, and use of health care services.^4,8,33,34,35,36^ However, few studies have evaluated the effect of CCTs on maternal mortality, with controversial results. A study conducted in Mexico^7^ reported an 11% decrease in maternal mortality, associated with higher *Oportunidades* CCT coverage levels. An investigation in India^8^ showed an increase in the percentage of births delivered at health facilities to be associated with being a beneficiary of the Indian CCT (JSY), but no effect on maternal mortality was found. The short period of the analysis following implementation of JSY (only 2 years) could be a limitation, since more time between the implementation and health service support may be required to affect maternal death among beneficiaries. An ecological study^9^ found a 17% decrease in maternal death in municipalities with higher BFP coverage over the last 11 years in Brazil.

The CCT BFP might affect maternal mortality through different mechanisms. First, the income transferred to women can have a more immediate effect, with the allocation of money for purchase of food, the use of health services, and mobility, particularly if it is coupled with shifts in intrahousehold power or autonomy favoring women.^37,38,39,40,41,42^ Second, by fulfilling conditionalities, BFP can increase access and the use of health services by reducing barriers and increasing the monitoring and treatment of comorbidities, facilitating referrals to high-risk prenatal care, and ensuring adequate assistance while giving birth. Receipt of BFP increased the interbirth interval and use of prenatal care, lending additional support to our hypothesis that BFP is associated with maternal mortality through the effect of relevant determinants of maternal mortality. In our study, an association of BFP with maternal mortality was found in municipalities with the highest level of FHP coverage. Conditional cash transfers are designed to have both short- and long-term effects on their beneficiaries’ lives.^43^ The findings of the present study suggest that the association between BFP and maternal mortality increased in magnitude, according to extended duration of the benefits. Studies have shown that longer exposure to a CCT program is associated with higher levels of engagement in utilizing health services^44^ and increased levels of education.^43,45^

Another important finding of our study is the association of BFP and maternal mortality among the most vulnerable (Black women, *Pardo *women, and women who live in rural areas and less developed municipalities), suggesting a potential contribution of BFP to reducing social inequalities.^46^ A high focalization of CCTs contributes to a significant downturn in susceptibilities among the most vulnerable beneficiaries. For example, Black women are disproportionately affected by poverty and maternal morbidities and mortality.^47,48^ Many of these morbidities, such as hypertension and gestational diabetes, can be controlled with adequate access to health care through conditionalities and receipt of the benefit, reducing access barriers and unmet needs.^49^ In addition, the most vulnerable women are more dependent on publicly provided and funded services,^49,50^ and the FHP was also prioritized within the more impoverished areas. The role of the FHP in the reduction of social inequalities has been well documented in the literature.^49,51,52,53^ Despite differences in the 2 public policies (social and health policies), it seems plausible that the mechanisms underlying the effects are synergic.

### Limitations

This study has some limitations. Although the 100 Million Brazilian Cohort is a powerful source of sociodemographic information, it contains data on the poorest half of the population; therefore, the results are not necessarily representative of all Brazilian women. Furthermore, residual confounding is possible, since data on other maternal mortality–related factors, including access to health care at the time of delivery, distance to services, the skills and preparedness of health care professionals, and preexisting comorbidities, were not available in the routinely collected data sets. We have attempted to minimize this by using different analytical approaches and performing subgroup and sensitivity analyses to strengthen the evidence produced and to minimize uncontrolled confounding. Limitations also arise from the linkage process (missed or false matches). However, we obtained a mean sensitivity and specificity of more than 92% in the linkage validation process, and these errors are probably nondifferential (eFigure 3 and eTable 18 in Supplement 1).

## Conclusions

The results of our cohort study show that a CCT program can substantially reduce maternal mortality in a large middle-income country such as Brazil. We have provided new evidence that continual exposure to BFP is associated with a reduction in maternal mortality, suggesting a long-term effect of CCTs. In the context of the COVID-19 pandemic and political instability affecting health care provision in Brazil, a country with considerable mortality among pregnant and postpartum women,^54,55,56,57^ we should consider intensifying efforts to maintain and implement appropriate social policies, in conjunction with improved quality of prenatal and obstetric care in the post–COVID-19 recovery period.

## Notes

Supplement 1. **eMethods.** Study Population, Propensity Score and Kernel Weighting, Sensitivity and Subgroup Analyses, and Data Linkage and Quality Assessment **eTable 1.** Description of Women Excluded From the Main Analysis Following Definition of *Bolsa Família* Program (BFP) Exposure **eTable 2.** Logistic Models to Estimate the Propensity Scores for *Bolsa Família* Participation **eTable 3.** ATT of Maternal Mortality for BFP Receipt Between 2004 and 2015 Using Kernel Matching **eFigure 1.** Propensity Scores Common Support Area **eFigure 2.** Cumulative Distribution Balancing Plot **eTable 4.** Baseline Characteristics of *Bolsa Família* Program (BFP) Nonbeneficiaries (non-BFP) and Beneficiaries From *Cadastro Único* Registration Between 2004 and 2015 **eTable 5.** Kernel-Weighted Regression and Other Strategies for Associations Between BFP Receipt and Maternal Death in 100 Million Brazilian Cohort, 2004-2015 **eTable 6.** Kernel-Weighted Regression for Associations Between BFP Receipt and Maternal Death in 100 Million Brazilian Cohort, 2004-2015 **eTable 7.** Kernel-Weighted Regression for Associations Between Duration of BFP Receipt and Maternal Death per Year in 100 Million Brazilian Cohort, 2004-2015 **eTable 8.** Kernel-Weighted Regression for Associations Between Length of Exposure to Receiving BFP and Maternal Death per Year in 100 Million Brazilian Cohort, 2004-2015 **eTable 9.** Kernel-Weighted Regression for Associations Between Length of Exposure to Receiving BFP/Age and Maternal Death per Year in 100 Million Brazilian Cohort, 2004-2015 **eTable 10.** Kernel-Weighted Regression for Associations Between Length of Exposure to Receiving BFP and Maternal Death per Year in 100 Million Brazilian Cohort, 2004-2015 **eTable 11.** Regression Results: Coefficients on Unadjusted Kernel-Weighted Logistic Regressions Within Subgroups of Municipal Human Development Index (MHDI) **eTable 12.** Regression Results: Coefficients on Unadjusted Kernel-Weighted Logistic Regressions Within Subgroups of Maternal Race **eTable 13.** Regression Results: Coefficients on Unadjusted Kernel-Weighted Logistic Regressions Within Subgroups of Area of Residence (Rural and Urban) **eTable 14.** Regression Results: Coefficients on Unadjusted Kernel-Weighted Logistic Regressions Within Subgroups of Municipal Quintiles of Family Health Program Coverage **eTable 15.** Logistic Models to Estimate the Propensity Scores for *Bolsa Família* Participation **eTable 16.** Average Treatment Effect on the Treated (ATT) Estimated Using Kernel-Matching (Excluding Type of Water Supply, Waste Disposal System [Sewer System], and Location of Household of the PS) **eTable 17.** Kernel-Weighted Regression for Associations Between BFP Receipt and Maternal Death (Excluding Type of Water Supply, Waste Disposal System [Sewer System], and Location of Household of the PS) in 100 Million Brazilian Cohort, 2004-2015 **eTable 18.** Accuracy Analysis of the Linkage Between *Cadastro Único* and the Mortality Information System in a Sample of 10 000 Record Pairs **eFigure 3.** ROC Curve of the Linkage Between Mortality and *Cadastro Único* Between 2001 and 2015
eReferences

Supplement 2.
Data Sharing Statement
