# Supplementary material for: Association of Conditional Cash Transfers With Maternal Mortality Using the 100 Million Brazilian Cohort
Source: JAMA Netw Open. 2023 Feb 23;6(2):e230070. doi: 10.1001/jamanetworkopen.2023.0070 (PMC9951038; doi:10.1001/jamanetworkopen.2023.0070)
Supplement: Supplement 1. — eMethods. Study Population, Propensity Score and Kernel Weighting, Sensitivity and Subgroup Analyses, and Data Linkage and Quality Assessment eTable 1. Description of Women Excluded From the Main Analysis Following Definition of Bolsa Família Program (BFP) Exposure eTable 2. Logistic Models to Estimate the Propensity Scores for Bolsa Família Participation eTable 3. ATT of Maternal Mortality for BFP Receipt Between 2004 and 2015 Using Kernel Matching eFigure 1. Propensity Scores Common Support Area eFigure 2. Cumulative Distribution Balancing Plot eTable 4. Baseline Characteristics of Bolsa Família Program (BFP) Nonbeneficiaries (non-BFP) and Beneficiaries From Cadastro Único Registration Between 2004 and 2015 eTable 5. Kernel-Weighted Regression and Other Strategies for Associations Between BFP Receipt and Maternal Death in 100 Million Brazilian Cohort, 2004-2015 eTable 6. Kernel-Weighted Regression for Associations Between BFP Receipt and Maternal Death in 100 Million Brazilian Cohort, 2004-2015 eTable 7. Kernel-Weighted Regression for Associations Between Duration of BFP Receipt and Maternal Death per Year in 100 Million Brazilian Cohort, 2004-2015 eTable 8. Kernel-Weighted Regression for Associations Between Length of Exposure to Receiving BFP and Maternal Death per Year in 100 Million Brazilian Cohort, 2004-2015 eTable 9. Kernel-Weighted Regression for Associations Between Length of Exposure to Receiving BFP/Age and Maternal Death per Year in 100 Million Brazilian Cohort, 2004-2015 eTable 10. Kernel-Weighted Regression for Associations Between Length of Exposure to Receiving BFP and Maternal Death per Year in 100 Million Brazilian Cohort, 2004-2015 eTable 11. Regression Results: Coefficients on Unadjusted Kernel-Weighted Logistic Regressions Within Subgroups of Municipal Human Development Index (MHDI) eTable 12. Regression Results: Coefficients on Unadjusted Kernel-Weighted Logistic Regressions Within Subgroups of Maternal Race eTable 13. Regression Results: Coe [file jamanetwopen-e230070-s001.pdf]

Supplementary Online Content

Alves FJO, Ramos D, Paixão ES, et al. Association of conditional cash transfers with maternal mortality using the 100 Million Brazilian Cohort. *JAMA Netw Open*. 2023;6(2):e230070. doi:10.1001/jamanetworkopen.2023.0070

eMethods. Study Population, Propensity Score and Kernel Weighting, Sensitivity and Subgroup Analyses, and Data Linkage and Quality Assessment

eTable 1. Description of Women Excluded From the Main Analysis Following Definition of *Bolsa Família* Program (BFP) Exposure

eTable 2. Logistic Models to Estimate the Propensity Scores for *Bolsa Família* Participation

eTable 3. ATT of Maternal Mortality for BFP Receipt Between 2004 and 2015 Using Kernel Matching

eFigure 1. Propensity Scores Common Support Area

eFigure 2. Cumulative Distribution Balancing Plot

eTable 4. Baseline Characteristics of *Bolsa Família* Program (BFP) Nonbeneficiaries (non-BFP) and Beneficiaries From *Cadastro Único* Registration Between 2004 and 2015

eTable 5. Kernel-Weighted Regression and Other Strategies for Associations Between BFP Receipt and Maternal Death in 100 Million Brazilian Cohort, 2004-2015

eTable 6. Kernel-Weighted Regression for Associations Between BFP Receipt and Maternal Death in 100 Million Brazilian Cohort, 2004-2015

eTable 7. Kernel-Weighted Regression for Associations Between Duration of BFP Receipt and Maternal Death per Year in 100 Million Brazilian Cohort, 2004-2015

eTable 8. Kernel-Weighted Regression for Associations Between Length of Exposure to Receiving BFP and Maternal Death per Year in 100 Million Brazilian Cohort, 2004-2015

eTable 9. Kernel-Weighted Regression for Associations Between Length of Exposure to Receiving BFP/Age and Maternal Death per Year in 100 Million Brazilian Cohort, 2004-2015

eTable 10. Kernel-Weighted Regression for Associations Between Length of Exposure to Receiving BFP and Maternal Death per Year in 100 Million Brazilian Cohort, 2004-2015

eTable 11. Regression Results: Coefficients on Unadjusted Kernel-Weighted Logistic Regressions Within Subgroups of Municipal Human Development Index (MHDI)

eTable 12. Regression Results: Coefficients on Unadjusted Kernel-Weighted Logistic Regressions Within Subgroups of Maternal Race

eTable 13. Regression Results: Coefficients on Unadjusted Kernel-Weighted Logistic Regressions Within Subgroups of Area of Residence (Rural and Urban)

eTable 14. Regression Results: Coefficients on Unadjusted Kernel-Weighted Logistic Regressions Within Subgroups of Municipal Quintiles of Family Health Program Coverage

eTable 15. Logistic Models to Estimate the Propensity Scores for *Bolsa Família* Participation

eTable 16. Average Treatment Effect on the Treated (ATT) Estimated Using Kernel-Matching (Excluding Type of Water Supply, Waste Disposal System [Sewer System], and Location of Household of the PS)

© 2023 Alves FJO et al. *JAMA Network Open*.

eTable 17. Kernel-Weighted Regression for Associations Between BFP Receipt and Maternal Death (Excluding Type of Water Supply, Waste Disposal System [Sewer System], and Location of Household of the PS) in 100 Million Brazilian Cohort, 2004-2015

eTable 18. Accuracy Analysis of the Linkage Between *Cadastro Único* and the Mortality Information System in a Sample of 10 000 Record Pairs

eFigure 3. ROC Curve of the Linkage Between Mortality and *Cadastro Único* Between 2001 and 2015

eReferences

This supplementary material has been provided by the authors to give readers additional information about their work.

eMethods. Study Population, Propensity Score and Kernel Weighting, Sensitivity and Subgroup Analyses, and Data Linkage and Quality Assessment

1. **The 100 Million Brazilian Cohort**

The 100 Million Brazilian Cohort baseline is an open cohort using data linkage compiled by the Centre

© 2023 Alves FJO et al. *JAMA Network Open*.

of Data and Knowledge Integration for Health (CIDACS/FIOCRUZ). The cohort is based on the notion of a "cohort baseline" with information on over 114 million individuals who were registered on the Brazilian National Registry for Social Programmes – Cadastro Único (CadÚnico) between 2001 and 2015. The eligibility criteria for CadÚnico registration includes a monthly family income per capita of half the minimum salary in Brazil (e.g. BRL 778.00 in 2015), or a total family income (monthly) of up to three minimum salaries[1]. It contains information on housing conditions, income, and demographic characteristics, and socioeconomic information on all members of a registered family. For those selected to receive Bolsa Família (BFP), information on BFP receipt was obtained through linking the cohort baseline with the BFP payroll database (2004-2015), which contains information on sociodemographic characteristics (i.e., date of birth and family income), start of receipt, and length of time receiving BFP.

1.1 Bolsa Família Programme (BFP)

The Brazilian cash transfer programme, BFP, is the flagship and largest socioeconomic programme implemented by the Brazilian government in 2004. It forms part of the Brazilian initiative to eradicate extreme poverty and has three objectives: an income guarantee for the immediate relief of extreme poverty; access to public services (improving the education, health, and citizenship of families); and productive inclusion to increase capacity, job opportunities, and income generation among the poorest families[1]. Implementation of BFP has resulted in 22.2 million Brazilians overcoming extreme poverty.

Every BFP participant is registered on CadÚnico. BFP is available throughout all the regions of Brazil and is estimated as having over 90% coverage among those eligible in the country. BFP beneficiaries receive basic, variable benefits, with values that vary over time, according to extreme poverty (families with a monthly per capita income of up to BRL 89.00), and poverty cut-off points (families with a monthly per capita income of up to BRL 178.00). The basic benefit is currently BRL 89.00 for extremely poor families. Variable benefits of BRL 41.00 are allocated to families with children, pregnant/puerperal women, or adolescents[1]. The BFP uses educational and health-related conditionalities to promote behavioural change. The conditionalities are the requirement that all children must attend a minimum of 85% of school days, and women and children must attend health care appointments. These conditionalities are based on the idea that making benefits conditional upon 'positive' behaviours can further increase the chance of families breaking out of the cycle of poverty through increased education, or improved health. For instance, increasing school attendance, and consequently improving educational levels, can also lead to improving the quality of social networks, i.e. making friends at school rather than on the streets, and reducing opportunities for certain types of crime and risky behaviour.

In our study, we have considered those exposed to BFP as women with records of live births who started to receive BFP before or during pregnancy, and continued to receive the benefit until childbirth, or before death. Women who had not received the benefit at any time, or until childbirth, were considered not exposed. The women who stopped receiving the benefit at some point before childbirth were excluded from the analysis. The characteristics of the population excluded from the analysis due to the definition of BFP exposure (women who stopped receiving the benefit at some point before childbirth) are described in Supplementary eTable 1.

eTable 1. Description of Women Excluded From the Main Analysis Following Definition of *Bolsa Família* Program (BFP) Exposure

**Socioeconomic and demographic variables**	**Population following definition of BFP exposure[1]**				**Women excluded following definition of BFP exposure[2]**
	**Non-BFP** n= 1,264,037	**BFP** n=5,413,236	**Total** n=6,677, 273		**Total** **N=**1,234,794
**Sociodemographic**					
Race/Colour					
Asian	0.47	0.35	0.37		0.30
Black	7.36	8.78	8.52		6.90
Indigenous	0.29	1.03	0.90		0.34
Mixed-race/brown	55.69	63.69	62.23		52.31
White	36.19	26.15	27.98		40.13
*Missing data**	9.35	5.76	5.23		5.53
Education					

High school/College (>=8 years)	69.64	58.88	60.92		78.24
Elementary/Middle school (4-7 years)	24.21	32.86	31.22		19.23
Elementary school or illiterate (<3 years)	6.15	8.26	7.86		2.53
*Missing data**	1.98	2.26	2.21		1.57
Age Groups					
10-19 years old	14.09	23.24	70.57		22.19
20-34 years old	75.89	69.32	21.51		70.53
>=35 years old	10.02	7.44	7.92		7.28
*Missing data**	0.0	0.0			0.0
Parity					
1 child in the cohort	39.74	44.35	43.48		34.24
2-3 children in the cohort	57.80	49.66	51.20		62.85
>3 children in the cohort	2.46	5.99	5.32		2.91
*Missing data**	0.0	0.0	0.0		0.0
**Household**					
Density					
<=2 per room	79.30	53.22	57.88		58.29
>2 per room	20.70	46.78	42.12		41.71
*Missing data**	11.63	5.10	6.33		5.37
Water Supply					
Public network	77.51	65.42	67.62		76.60
Well/natural source/other	22.49	34.58	32.38		23.40
*Missing data**	7.53	2.79	3.69		3.28
Waste disposal system					
Public network	77.51	38.72	40.97		51.34
Septic tank/ditch/other	22.49	61.28	59.02		48.65
*Missing data**	8.88	3.51	4.52		3.46
Garbage disposal					
Public collection system	51.21	68.78	71.19		81.97
Burned/buried/other	49.79	31.22	28.81		18.03
*Missing data**	7.53	2.79	3.69		3.28
**Geographical**					
Region					
South	14.19	8.55	9.61		16.47
North	9.78	13.21	12.56		8.73
Northeast	30.69	42.87	40.57		22.09
Southeast	35.10	29.29	30.39		43.46
Central-West	10.24	6.08	6.86		9.23
*Missing data**	0	0.0	0.0		0.0
Household location					
Urban	81.56	71.74	73.55		82.69
Rural	18.44	28.26	26.45		17.31
*Missing data**	5.56	2.06	2.72		2.75
**Year**					
2004	7.85	10.26	9.80		10.26
2005	7.12	7.92	7.76		8.97
2006	26.84	45.62	42.06		53.97
2007	11.18	13.71	13.23		12.66
2008	5.37	5.53	5.50		4.10
2009	4.33	4.85	4.75		3.15

© 2023 Alves FJO et al. *JAMA Network Open*.

2010	6.12	4.36	4.70		2.96
2011	5.18	2.51	3.02		1.63
2012	10.41	2.79	4.23		1.21
2013	7.44	1.61	2.71		0.62
2014	6.09	0.72	1.73		0.31
2015	2.07	0.12	0.49		0.14
*Missing data**	0	0.0	0.0		0

[1]Main analysis population: BFP - women who started to receive the BFP before or during pregnancy, and continued to receive the benefit until childbirth, or before death. Non-BFP: Women who had not received the benefit at any time, or until childbirth.

[2] Excluded women - women who stopped receiving the benefit at some point before childbirth

2. Propensity score and kernel weighting

2.1 Propensity score estimation

BFP is allocated according to the criteria of poverty and extreme poverty, which is classified by per capita family income.[1] However, a set of socioeconomic characteristics are related to program receipt. Since as the receipt of the program was not randomly assigned to families, iIn line with our research protocol[2] and previous quasi-experimental studies using the CadUnico dataset and the 100 Million Brazilians Cohort, the association between BFP and maternal mortality was estimated based on the propensity score-based method (PS).

We estimated the probability of receiving the BFP benefit from the baseline covariates using multiple logistic regressions. The following covariates were considered to estimate the propensity score (PS): self-declared race/skin colour (white, mixed-race-brown, black, indigenous, or of Asian descent), level of education (≤3 years, 4-7 years, or ≥8 years), age (10-19 years old; 20-34 years old; and >=35 years old), parity (number of childbirths in the cohort: 1; 2-3 and >= 3 children), urban/rural residency), region (North, Northeast, Southeast, South and Central-West), household density (≤2 inhabitants per room, or >2 inhabitants per room), water supply (public network, or a well, natural source or other), waste disposal system (public network, septic tank, ditch, or other), garbage disposal (public collection system, burned, buried, or other) and year of registration on Cadastro Único. eTable 2 shows the estimates from the logit model for the probability of receiving BFP (propensity scores) based on women baseline characteristics and socioeconomic conditions.

eTable 2. Logistic Models to Estimate the Propensity Scores for *Bolsa Família* Participation

Variables	Odds Ratio (95% CI)	p-value	Standard Error
**Parity**			
1 child in the cohort	Ref.		-
2-3 children in the cohort	0.57 (0.56 - 0.57)	<0.001	0.0014
>3 children in the cohort	2.20 (2.17 - 2.23)	<0.001	0.0016
**Education**			

© 2023 Alves FJO et al. *JAMA Network Open*.

High school/College (>=8 years)	Ref		
Elementary/Middle school (4-7 years)	1.14 (1.13-1.14)	<0.001	0.0073
Elementary school or illiterate (<3 years)	0.91 (0.90-0.92)	<0.001	0.0038
**Age Groups**			
20-34	Ref.		-
≤ 19	2.09 (2.08 - 2.11)	<0.001	0.0073
≥ 35	0.92* (0.92- 0.93)	<0.001	0.0038
**Maternal race/skin color**			
White	Ref.		-
Asian descendants	1.15 (1.11 - 1.19)	<0.001	0.0218
Black	1.31 (1.30 - 1.33)	<0.001	0.0071
Indigenous	3.15 (3.02 - 3.29)	<0.001	0.0692
Mixed/brown	1.20 (1.19 - 1.21)	<0.001	0.0342
**Region**			
South	Ref.		-
North	1.49 (1.48-1.51)	<0.001	0.0082
Northeast	1.94 (1.92 - 1.96)	<0.001	0.0082
Southeast	1.47 (1.46 - 1.49)	<0.001	0.0607
Center-West	0.90 (0.89 - 0.91)	<0.001	0.0048
**Local domiclio**			
Urban	Ref.		-
Rural	0.99 (0.98 - 1.00)	<0.146	0.0043
**Household density**			

© 2023 Alves FJO et al. *JAMA Network Open*.

≤ 2 people per room	Ref.		-
> 2 people per room	2.44<br>(2.43-2.46)	<0.001	0.0684
**Water Supply**			
Public network	Ref.		-
Well/natural source/other	1.15<br>(1.14 - 1.16)	<0.001	0.0141
**Waste disposal system**			
Public network	Ref.	<0.001	-
Septic tank/ditch/other	1.88<br>(1.86 - 1.91)	<0.001	0.0406
**Garbage disposal**			
Public collection system			
Burned/buried/other	1.31<br>(1.29-1.32)	<0.001	0.0057
**Registration year in Cadastro Unico**			
**2004**	Ref		
**2005**	0.99<br>(0.98-1.00)	0.292	0.0058
**2006**	1.16<br>(1.15-1.17)	<0.001	0.0051
**2007**	0.78<br>(0.78-0.79)	<0.001	0.0039
**2008**	0.70<br>(0.69-0.71)	<0.001	0.0042
**2009**	0.81<br>(0.80-0.82)	<0.001	0.0051
**2010**	0.53<br>(0.53-0.54)	<0.001	0.0034
**2011**	0.37<br>(0.36-0.37)	<0.001	0.0027
**2012**	0.21<br>(0.21-0.22)	<0.001	0.0012
**2013**	0.18<br>(0.18-0.18)	<0.001	0.0013
**2014**	0.09<br>(0.09-0.09)	<0.001	0.0741

© 2023 Alves FJO et al. *JAMA Network Open*.

**2015**	0.45<br>(0.43-0.47)	<0.001	0.0072

2.2 Kernel matching/weighting

Kernel matching establishes a non-parametric relationship between the PS and outcome - maternal mortality, selecting observations of non-beneficiaries (non-BFP) who were similar to the set of treated groups (BFP beneficiaries), according to observable characteristics (PS).[6] There are weighting schemes for all of the untreated groups (non-BFP), and the weights depend on the distance between each individual from the control group, and the participant observation for which the counterfactual is estimated[7]. The basic idea behind kernel estimation is giving different weight to observations with a different distance.

We estimated the average treatment effect on the treated (ATT) by kernel matching (eTable 3). eFig 1 shows the distribution of estimated propensity scores given the covariates. Overlapping histograms are displayed for the beneficiaries and non-beneficiaries. eFig2 shows the balance between variables before and after Kernel matching.

eTable 3. ATT of Maternal Mortality for BFP Receipt Between 2004 and 2015 Using Kernel Matching

	**Kernel Weighting**
	**ATT* (95%CI)**
ATT	-0.0001096 (-0.0002052, -0.0000139)
N	5,757,188

*Average treatment effect on the treated (ATT) estimated using kernel matching (PS variables).

© 2023 Alves FJO et al. *JAMA Network Open*.

eFigure 1. Propensity Scores Common Support Area

Distribution of propensity scores across beneficiaries and non-beneficiaries

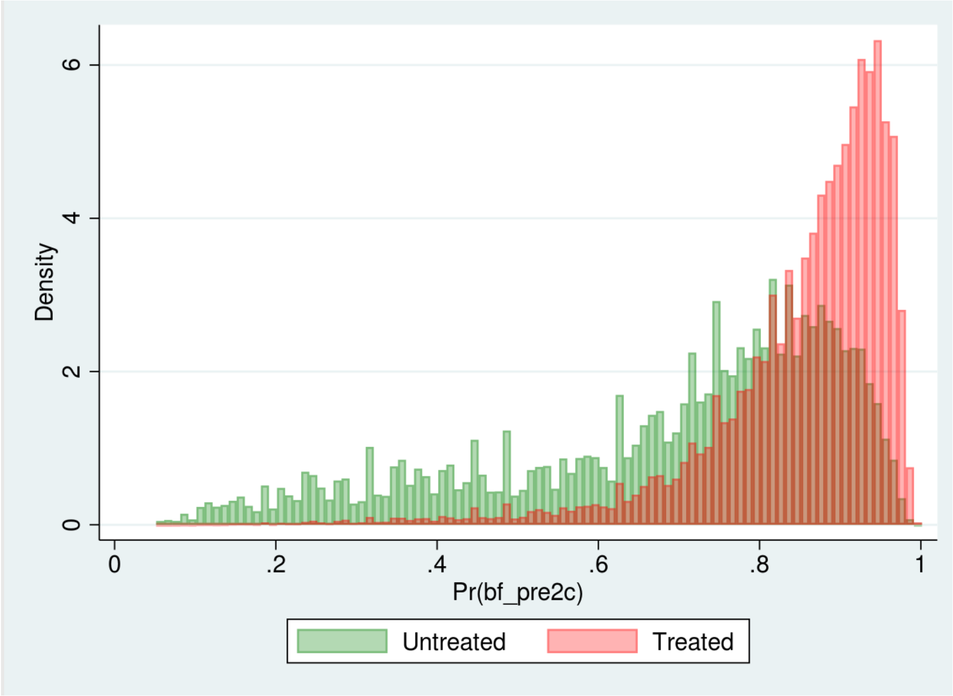

eFigure 2. Cumulative Distribution Balancing Plot

Balance between variables before and after Kernel matching]

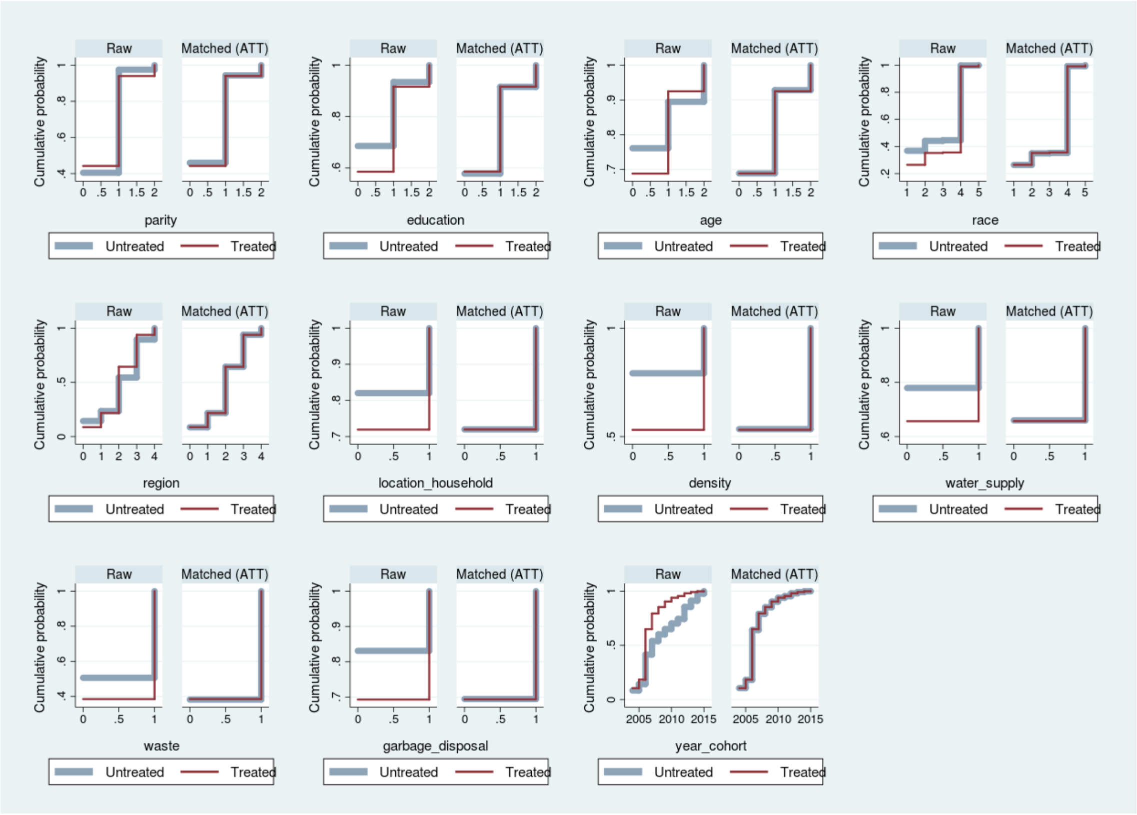

© 2023 Alves FJO et al. *JAMA Network Open*.

3. Sensitivity analysis

3.1 **Inverse probability of treatment weighting (IPTW)**

We used the same framework of analysis for the kernel weighting, to estimate the effect of treatment on the treated (ATT) using weights. First, we estimated the propensity score (PS) for receiving BFP from the cohort baseline sociodemographic covariates. Second, we estimated the weights for BFP beneficiary (weight=1) and non-BFP beneficiary families (weight= E(PS)/(1-E(PS)). We estimated the logistic regression using inverse probability of treatment weighting (IPTW) and compared the differences in the distribution of PS covariates between beneficiaries and non-beneficiaries using proportions, to assess the balance of potential confounders before and after IPTW (eTable 4). This process yielded very similar results to the kernel approach, with similar rates with the IPTW balance (61.41 (59.35-63.53) and 70.67 (62.48-79.92)) for beneficiaries and non-beneficiaries (non-BFP), respectivelly (eTable 5). We also performed unweighted multivariate logistic regression and tbeneficiaries were slightly higher than non-beneficiaries (61.41(59.35-63.56) and 57.91 (53.86-62.26)). Adjusted IPTW results (weighted $_{OR}$:0.84, 95%CI:0.74-0.96) and unweighted multivariate logistic regression (OR:0.89, 95%CI:0.81-0.98) analysis, consistently showed a similar magnitude of associations (eTable 5).

© 2023 Alves FJO et al. *JAMA Network Open*.

eTable 4. Baseline Characteristics of Bolsa Família Program (BFP) Nonbeneficiaries (non-BFP) and Beneficiaries From *Cadastro Único* Registration Between 2004 and 2015

**Socioeconomic and demographic variables**	**Before IPTW**			**After IPTW**		
**Sociodemographic**	**Non-BFP** n= 1,264,037	**BFP** n=5,413,236	**Diff (BFP-Non-BFP)**	**Non-BFP** n=1,020,639	**BFP** n=5,413,236	**Diff (BFP-Non-BFP)**
Race/Colour						
Asian	0.47	0.35	-0.12	0	0	0
Black	7.36	8.78	1.42	9.04	8.90	-0.14
Indigenous	0.29	1.03	0.74	0	0	0
Mixed-race	55.69	63.69	8	64.63	64.58	-0.05
White	36.19	26.15	-10.04	26.33	26.52	0.19
Education						
High school/College (>=8 years)	69.64	58.88	-10.76	54.81	58.88	4.07
Elementary/Middle school (4-7 years)	24.21	32.86	8.65	35.59	32.86	-2.73
Elementary school or illiterate (<3 years)	6.15	8.26	2.11	9.59	8.26	-1.33
Age Groups						
10-19 years old	14.09	23.24	9.15	22.42	23.24	0.82
20-34 years old	75.89	69.32	-6.57	70.19	69.32	-0.87
>=35 years old	10.02	7.44	-2.58	7.39	7.44	0.05

© 2023 Alves FJO et al. *JAMA Network Open*.

**Sociodemographic**		**Non-BFP** n= 1,264,037	**BFP** n=5,413,236	**Diff (BFP-Non-BFP)**		**Non-BFP** n=1,020,639	**BFP** n=5,413,236	**Diff (BFP-Non-BFP)**
**Parity**								
1 child in the cohort		39.74	44.35	4.61		44.35	44.35	0
2-3 children in the cohort		57.80	49.66	-8.14		49.66	49.66	0
>3 children in the cohort		2.46	5.99	3.53		5.99	5.99	0
**Household**								
Density								
<=2 per room		79.30	53.22	-26.08		53.56	53.22	-0.34
>2 per room		20.70	46.78	26.08		46.44	46.78	0.34
Water Supply								
Public network		77.51	65.42	-12.09		65.36	65.42	0.06
Well/natural source/other		22.49	34.58	12.09		34.64	34.58	-0.06
Waste disposal system								
Public network		77.51	38.72	-38.79		38.71	38.72	0.01
Septic tank/ditch/other		22.49	61.28	38.79		61.29	61.28	-0.01
Missing data		8.88	3.51				3.51	3.51
Garbage disposal								
Public collection system		51.21	68.78	17.57		69.39	68.78	-0.61

© 2023 Alves FJO et al. *JAMA Network Open*.

Burned/buried/other		49.79	31.22	-18.57		30.61	31.22	0.61
**Geographical**								
Region								
South		14.19	8.55	-5.64		8.43	8.55	0.12
North		9.78	13.21	3.43		13.52	13.21	-0.31
Northwest		30.69	42.87	12.18		41.19	42.87	1.68
Southwest		35.10	29.29	-5.81		30.22	29.29	-0.93
Central-West		10.24	6.08	-4.16		6.64	6.08	-0.56
Household location								
Urban		81.56	71.74	-9.82		72.07	71.74	-0.33
Rural		18.44	28.26	9.82		27.93	28.26	0.33
**Year**								
2004		7.85	10.26	2.41		11.00	10.26	-0.74
2005		7.12	7.92	0.8		7.70	7.92	0.22
2006		26.84	45.62	18.78		44.19	45.62	1.43
2007		11.18	13.71	2.53		15.55	13.71	-1.84
2008		5.37	5.53	0.16		6.41	5.53	-0.88
2009		4.33	4.85	0.52		5.28	4.85	-0.43
2010		6.12	4.36	-1.76		3.65	4.36	0.71
2011		5.18	2.51	-2.67		1.56	2.51	0.95
2012		10.41	2.79	-7.62		2.61	2.79	0.18

© 2023 Alves FJO et al. *JAMA Network Open*.

2013		7.44	1.61	-5.83		1.21	1.61	0.4
2014		6.09	0.72	-5.37		0.71	0.72	0.01
2015		2.07	0.12	-1.95		0.13	0.12	-0.01

eTable 5. Kernel-Weighted Regression and Other Strategies for Associations Between BFP Receipt and Maternal Death in 100 Million Brazilian Cohort, 2004-2015

	**Kernel Weighting**						**IPTW**						**Unweighted Logistic Regression**				
	**Weighted Rates**			**OR(95%IC)**			**Weighted Rates**			**OR(95%IC)**			**Rates**			**OR(95%IC)**	
	**Non-BFP[a]**	**BFP[a]**		**OR**	**Adjusted OR[b]**		**Non-BFP[a]**	**BFP[a]**		**OR**	**Adjusted OR[b]**		**Non-BFP[a]**	**BFP[a]**		**OR**	**Adjusted OR[c]**
Rates /OR	71.59 (62.87-81.53)	60.63 (58.46-62.89)		0.84 (0.73-0.96)	0.82 (0.71-0.93)		70.67 (62.48-79.92)	61.41 (59.35-63.53)		0.87 (0.76-0.98)	0.84 (0.74-0.96)		57.91 (53.86-62.26)	61.41 (59.35-63.56)		0.91 (0.83-1.00)	0.89 (0.81-0.98)
n	1,017,154	4,731,624		5,748,917	5,542,230		1,020,639	5,413,236		6,433,875	6,175,248		1,264,037	5,413,236		5,879,995	5,665,567

[a]Non-BFP and BFP correspond to maternal rates/100,000 parturients of SINASC to non-beneficiaries and beneficiaries, respectivelly

[b]Adjusted by prenatal care, gestational age, type of delivery, and multiple pregnancy

[c] Multivariate logistic regression adjusted by PS variables.

© 2023 Alves FJO et al. *JAMA Network Open*.

3.2 **Varying definitions of exposure**

In order to verify whether different definitions of BFP exposure could affect the results, we fitted the same kernel weighting procedure with varying exposure definitions, obtaining similar findings (eTables 6 and 7). In the main strategy (main document), we considered as exposed the BFP beneficiary women with records of children born alive who started to receive BFP before or during pregnancy, and continued to receive the benefit before the outcome, or until childbirth. Unlike our main exposure, we only consider as unexposed those who did not receive BFP at any time in the entire cohort (eTable 6). The results are similar to the main definition of non-exposed (women who did not receive the benefit at any time, or until childbirth, were considered not exposed) (eTable 6).

We also verified other definitions considering the period of pregnancy covered by the exposure. Thus, we considered as exposed the BFP beneficiary women with records of children born alive who started to receive PBF, 22 weeks (foetal viability proxy), 6, or 9 months before childbirth. The results were similar to those found in the main analysis (eTable 7).

© 2023 Alves FJO et al. *JAMA Network Open*.

eTable 6. Kernel-Weighted Regression for Associations Between BFP Receipt and Maternal Death in 100 Million Brazilian Cohort, 2004-2015[1]

Kernel weighting						Unweighted estimates				
Kernel weighted rates by 100,000 SINASC parturients			Kernel weighted logistic regression			Rates by 100,000 SINASC parturients			Unweighted logistic regression	
Non-BFP	BFP		OR (95%CI)	Adjusted OR (95%CI)[2]		Non-BFP (95%CI)	BFP (95%CI)		OR (95%CI)[3]	Adjusted OR (95%CI)[2]
62.56<br>(56.58-69-10)	61.41<br>(59.35-63.53)		0.56<br>(0.42, 0.75)	0.58<br>(0.44, 0.78)		107.19<br>(81.04-141.75)	60.47<br>(58.29-62.72)		0.79<br>(0.69, 0.90)	0.82<br>(0.71, 0.95)
n=609,049	n=4,720,036		n=5,185,459	n=4,992,866		n=465423	n=4,720,036		n=5,204,589	n=5,011,102

[1] Exposed: BFP beneficiary women with records of children born alive who started to receive PBF before or during pregnancy, and continued to receive the benefit before the outcome or until childbirth; Not Exposed: Did not receive BFP at any time in the entire cohort.

[2] Adjusted by prenatal care, gestational age, type of delivery and multiple pregnancy.

[3] Multivariate logistic regression adjusted by PS variables.

eTable 7. Kernel-Weighted Regression for Associations Between Duration of BFP Receipt and Maternal Death per Year in 100 Million Brazilian Cohort, 2004-2015

	22 weeks before childbirth		6 months before childbirth		9 months before childbirth
	OR (95%CI)		OR (95%CI)		OR (95%CI)
OR	0.83(0.72-0.95)		0.82(0.71-0.94)		0.83(0.72-0.96)
N	5,498,605		5,459,798		5,325,764

[1]Exposed: BFP beneficiary women with records of children born alive who started to receive BFP 22 weeks (foetal viability proxy), 6, or 9 months before childbirth; Not Exposed: women who did not receive the benefit at any time, or until childbirth.

© 2023 Alves FJO et al. *JAMA Network Open*.

3.3 Length of exposure to receipt of Bolsa Família

As described in the main text, in order to evaluate BFP duration effects, we fitted the same kernel weighting procedure with varying exposure definitions, comparing women exposed to different ranges of years between the BFP receipt until delivery (1-4 years, 5-8 years, or ≥9 years), with women not exposed to the programme. Additionally, we calculated an indicator considering the years of exposure to the BFP, divided by the woman's age on delivery, multiplied by 100, resulting in a proxy of the percentage of the life of women exposed to PBF until delivery (mean=30.44%), classified as <30%, 30 to 70%, and > = 70%. The results for the analysis with varying exposure definitions can be found on eTables 8 and 9.

eTable 8. Kernel-Weighted Regression for Associations Between Length of Exposure to Receiving BFP and Maternal Death per Year in 100 Million Brazilian Cohort, 2004-2015[1]

	**Length of exposure to receipt of Bolsa Família**		
	**1-4 years***	**5-8 years***	**>= 9 years***
**Weighted Odds Ratio***	0.85	0.70	0.68
**(95% CI)**	0.75-0.97	0.60-0.82	0.53-0.88
**N**	5,220,204	2,189,711	1,071,053

[1]All the analytical steps (PS estimation, kernel matching and weighted logistic regressions) were conducted separately considering varying exposure definitions: Exposed- BFP beneficiary women who started to receive PBF for 1-4, 5-8, or >=9 years before childbirth; Not exposed: women who did not receive the benefit at any time, or until childbirth.

eTable 9. Kernel-Weighted Regression for Associations Between Length of Exposure to Receiving BFP/Age and Maternal Death per Year in 100 Million Brazilian Cohort, 2004-2015[1]

	**Length of exposure to receipt of Bolsa Família/age**		
	**<30%**	**30-70%**	**>=70%**
**OR**	0.92	0.52	0.39
**CI95%**	0.77-1.09	0.40-0.69	0.18-0.82
**N**	3,256,101	4,650,341	1,298,905

All the analytical steps (PS estimation, kernel matching and weighted logistic regressions) were conducted separately considering varying exposure definitions: Exposed: years of exposure of the BFP, divided by the woman's age on delivery, multiplied by 100, resulting in a proxy of the percentage of the life of women exposed to PBF until delivery(<30, 30-70, >=70) ; Not exposed: women who did not receive the benefit at any time, or until childbirth.

© 2023 Alves FJO et al. *JAMA Network Open*.

In order to test the robustness of the analysis by length of exposure, we estimated kernel matching and weighted logistic regressions for each year of duration of BFP receipt in the study period. There was a dose-response association with BFP and maternal mortality until the 8th year of exposure, confirming the increase in association over time found in the main analysis (eTable 10).

© 2023 Alves FJO et al. *JAMA Network Open*.

eTable 10. Kernel-Weighted Regression for Associations Between Length of Exposure to Receiving BFP and Maternal Death per Year in 100 Million Brazilian Cohort, 2004-2015[1]

	**1 year**	**2 years**	**3 years**	**4 years**	**5 years**	**6 years**	**7 years**	**8 years**	**9 years**	**10 years**	**11 years**
**OR**	0.80	0.78	0.74	0.70	0.67	0.62	0.60	0.56	0.57	0.57	0.34
**CI95%**	0.70-0.93	0.68-0.91	0.64-0.87	0.59-0.82	0.56-0.80	0.52-0.76	0.49-0.73	0.45-0.70	0.43-0.76	0.33-0.98	0.10-1.13
**N**	5,186,550	4,056,807	3,466,353	3,458,245	2,914,401	2,334,259	1,778,948	1,216,999	646,838	196,083	93,616

[1]Exposed: BFP beneficiary women with records of children born alive who started to receive PBF for 1, 2, 3, 4, 5, 6, 7, 8, 9, 10, or 11 years before childbirth; Not exposed: women who did not receive the benefit at any time, or until childbirth.

© 2023 Alves FJO et al. *JAMA Network Open*.

3.4 Subgroup analysis

To justify our hypothesis that the association between BFP and maternal mortality varies across subgroups, we conducted an analysis stratified by subgroups of some sociodemographic indicators, such as race (white, black/mixed black), area of residence (rural, urban) and Municipal Human Development Index – MHDI (high or very high, medium, low, very low). We estimated the propensity score for each subgroup of these sociodemographic indicators, with the same variables as the previous steps, as well as conducted kernel-weighted logistic models separately within each subgroup (eTables 11-14).

eTable 11. Regression Results: Coefficients on Unadjusted Kernel-Weighted Logistic Regressions Within Subgroups of Municipal Human Development Index (MHDI)[1]

Municipal Human Development Index – MHDI	**Weighted Odds Ratio*** **(95% CI)**	**Robust Standard Error**	**p-value**	**N**
**Model c1**				
High or very high	1.01 (0.85 to 1.20)	0.004	0.510	3,149,053
Constant	0.002 (0.001 to 0.002)	0.0001	< 0.001	
**Model c2**				
Medium	0.94 (0.76 to 1.17)	0.055	0.053	2,062,570
Constant	0.002 (0.002 to 0.003)	0.0001	< 0.001	
**Model c3**				
Low, very low	0.62 (0.47 to 0.84)	0.061	0.010	1,465,433
Constant	0.003 (0.003 to 0.004)	0.0002	< 0.001	

* Beneficiary status (BFP=1)

[1] All the analytical steps (PS estimation, kernel matching and weighted logistic regressions) were conducted separately for each category of this subgroup.

© 2023 Alves FJO et al. *JAMA Network Open*.

eTable 12. Regression Results: Coefficients on Unadjusted Kernel-Weighted Logistic Regressions Within Subgroups of Maternal Race[1]

Race	Weighted Odds Ratio* (95% CI)	Robust Standard Error	p-value	N
**Model d1**				
White	0.98 (0.68 to 1.42)	0.183	0.942	1,645,603
Constant	0.005 (0.003 to 0.007)	0.0001	< 0.001	
**Model d2**				
Black/ Mixed Black	0.79 (0.69 to 0.93)	0.064	< 0.001	4,126,296
Constant	0.007 (0.007 to 0.009)	0.0001	< 0.001	

* Beneficiary status (BFP=1)

[1] All the analytical steps (PS estimation, kernel matching and weighted logistic regressions) were conducted separately for each category of this subgroup.

© 2023 Alves FJO et al. *JAMA Network Open*.

eTable 13. Regression Results: Coefficients on Unadjusted Kernel-Weighted Logistic Regressions Within Subgroups of Area of Residence (Rural and Urban)[1]

**Area of residence**	**Weighted Odds Ratio*** **(95% CI)**	**Robust Standard Error**	**p-value**	**N**
**Model e1**				
Urban	0.89 (0.77 to 1.04)	0,183	0.160	4,262,349
Constant	0.006 (0.006 to 0.008)	0.005	< 0.001	
**Model e2**				
Rural	0.69 (0.53 to 0.92)	0,099	0.008	1,486,571
Constant	0.004 (0.003 to 0.005)	0.001	< 0.001	

* Beneficiary status (BFP=1)

[1]All the analytical steps (PS estimation, kernel matching and weighted logistic regressions) were conducted separately for each category of this subgroup.

© 2023 Alves FJO et al. *JAMA Network Open*.

eTable 14. Regression Results: Coefficients on Unadjusted Kernel-Weighted Logistic Regressions Within Subgroups of Municipal Quintiles of Family Health Program Coverage[1]

Family Health Programme Coverage	Weighted Odds Ratio* (95% CI)	Robust Standard Error	p-value	N
**Model b1**				
>=70%	0.76 (0.57 to 0.97)	0.205	0.001	3,294,584
Constant	0.007 (0.006 to 0.009)	0.0007	< 0.001	
**Model b2**				
30-70%	0.98 (0.78 to 1.22)	0.215	0.677	2,233,112
Constant	0.006 (0.005 to 0.008)	0.0006	< 0.001	
**Model b3**				
<=30%	1.04 (0.78 to 1.39)	0.147	0.757	1,134,887
Constant	0.007 (0.005 to 0.009)	0.0001	< 0.001	

* Beneficiary status (BFP=1)

[1] All the analytical steps (PS estimation, kernel matching and weighted logistic regressions) were conducted separately for each category of this subgroup.

© 2023 Alves FJO et al. *JAMA Network Open*.

3.5 Variables for matching

The selection of variables was guided by the literature and the availability of such variables in our dataset, considering factors related to both treatment and outcome, as suggested by Sturmes et al, 2014[8]; Austin, 2011[9], Brookhart et al, 2010[10], Ali, et al, 2019. The 100 Million Brazilian Cohort comprises the poorest half of the Brazilian population. Therefore, socioeconomic factors must be considered when estimating the PS, as they are important confounders of this relationship. However, household conditions variables, such as type of water supply, waste disposal system (sewer system) and location of household , could influence the BFP receipt, as well be impacted by the recipient of the benefit. This may violate the conditions necessary for these covariates to be considered exogenous. To reduce possible biases, We performed robustness analyses excluding water type, location of household (rural and urban), and waste type from the set of covariates used to match on for and we obtained similar results (OR:0,81 CI: 0.70-0.93) (eTable 15-17).

© 2023 Alves FJO et al. *JAMA Network Open*.

eTable 15. Logistic Models to Estimate the Propensity Scores for *Bolsa Família* Participation

Variables	Odds Ratio (95% CI)	p-value	Standard Error
**Parity**			
1 child in the cohort	Ref.		-
2-3 children in the cohort	0.57 (0.57- 0.58)	<0.001	0.0014
>3 children in the cohort	2.18 (2.15 - 2.11)	<0.001	0.0015
**Education**			
High school/College (>=8 years)	Ref		
Elementary/Middle school (4-7 years)	1.16 (1.15-1.16)	<0.001	0.0031
Elementary school or illiterate (<3 years)	0.91 (0.90-0.92)	<0.001	0.0045
**Age groups**			
20-34	Ref.	<0.001	-
≤ 19	2.06 (2.05 - 2.08)	<0.001	0.0071
≥ 35	0.90 (0.90- 0.92)	<0.001	0.0037
**Race**			
White	Ref.		-
Asian descent	1.16 (1.12 - 1.21)	<0.001	0.0216
Black	1.32 (1.31 - 1.34)	<0.001	0.0063
Indigenous	3.41 (3.27 - 3.55)	<0.001	0.0703
Mixed/Black	1.22 (1.21 - 1.23)	<0.001	0.0344
**Region**			
South	Ref.		-

© 2023 Alves FJO et al. *JAMA Network Open*.

North	1.66<br>(1.65-1.68)	<0.001	0.0089
Northeast	2.00<br>(1.98 - 2.01)	<0.001	0.0084
Southeast	1.42<br>(1.41 - 1.43)	<0.001	0.0057
Center-West	0.93<br>(0.92 - 0.94)	<0.001	0.0049
**Garbage disposal**			
Public collection system	Ref.		-
Burned/buried/other	1.36<br>(1.35 - 1.37)	<0.001	0.0866
**Registration year in Cadastro Unico**			
**2004**	Ref		
**2005**	1.00<br>(0.99-1.01)	0.647	0.0059
**2006**	1.74<br>(1.16-1.18)	<0.001	0.0052
**2007**	0.79<br>(0.78-0.80)	<0.001	0.0040
**2008**	0.70<br>(0.69-0.71)	<0.001	0.0041
**2009**	0.80<br>(0.80-0.81)	<0.001	0.0051
**2010**	0.53<br>(0.52-0.53)	<0.001	0.0034
**2011**	0.37<br>(0.37-0.38)	<0.001	0.0027
**2012**	0.21<br>(0.21-0.22)	<0.001	0.0012
**2013**	0.18<br>(0.18-0.18)	<0.001	0.0013
**2014**	0.09<br>(0.09-0.09)	<0.001	0.0070
**2015**	0.42<br>(0.42-0.44)	<0.001	0.0067

© 2023 Alves FJO et al. *JAMA Network Open*.

eTable 16. Average Treatment Effect on the Treated (ATT) Estimated Using Kernel-Matching (Excluding Type of Water Supply, Waste Disposal System [Sewer System], and Location of Household of the PS)

	**Kernel Weighting**
	**ATT* (95%CI)**
ATT	-0.0001275 (-0.0002252, -0.0000297)
N	5,879,643
*Average treatment effect on the treated (ATT) estimated using kernel matching (PS variables).	

eTable 17. Kernel-Weighted Regression for Associations Between BFP Receipt and Maternal Death (Excluding Type of Water Supply, Waste Disposal System [Sewer System], and Location of Household of the PS) in 100 Million Brazilian Cohort, 2004-2015

	**Weighted Odds Ratio*<br>(95% CI)**	
	0.81(0.70-0.93)	
N	5,662,191	

4. Data linkage and quality assessment

4.1 100 Million Brazilian Cohort baseline versus Mortality Information System linkage

The women who died by maternal causes in the cohort were identified by linking data from the Brazilian Mortality Information System (SIM) with the 100 Million Cohort dataset for a 15 year period (2000 to 2015). The 100 Million Cohort and SIM databases were linked by the Centre for Data and Knowledge Integration for Health (Barreto et al., 2019), using record linkage software developed by the centre. This section provides a detailed description of how this linkage was carried out.

© 2023 Alves FJO et al. *JAMA Network Open*.

The linkage algorithm used five variables to identify matching records from the two databases (SIM and 100 Million Cohort), and each one was recorded in both of the linked datasets: beneficiary's name, mother's name, municipality of residence code, and date of birth.

The record linkage software performed two main steps:

First, the record corresponding to data for each 100 Million Cohort individual (the larger dataset) was indexed in the Lucene Apache library[11]. Lucene Apache has a method of indexing files, and performed a data search in these files. After this initial structuring, the data was saved in an indexed document with an identification number. At the end of the process, a folder was generated with the indexed database.

Second, for each death in the SIM dataset, the algorithm searched the 100 Million cohort indexed database for a potential match. The variables used to match the individual records were: the beneficiary's name, mother's name, date of birth, municipality of residence, and sex. All of the records must include the beneficiary's name, in order to perform the linkage. The records which did not include the beneficiary's name were excluded. The software performs this search at three levels:

Level 1: A first search is made through all of the five selected variables. The mortality data (SIM) found in the 100 Million Cohort baseline is saved, generating a list of linked records. A similarity calculation is performed for the linked records, generating a score which indicates the similarity between the two linked records. The similarity calculation is performed by comparing names, dates of birth, states, municipalities, and sex for the two records (one in each dataset) of the linked individuals. This process generates a "weighted average," which is the value of the similarity score. If the score is equal to, or greater than 0.95, the link is accepted (as being a correct link).

Level 2: If the similarity calculation is less than 0.95, a new search is performed (this time only including those matched under the 0.95 score), using a group of four (of the five previous variables) per time, in five different combinations. Subsequently, all search results with each group of variables are pooled, and the similarity test is performed. Again, if the score is equal to, or greater than 0.95, the linked data is accepted.

Level 3: Lastly, if a SIM individual is not linked to a 100 Million Cohort record in the two previous searches, a final search is undertaken. For this search, the beneficiary's name, mother's name, municipality of residence code, and date of birth are divided into smaller pieces. For example, the date of birth is divided into three smaller categories: day, month, and year. Several attempts are made, to try to match the person in both datasets. In order to obtain confirmation on whether the record linked is actually valid, a distance-editing method is applied, based on Jaro-Winkler.

In order to check the entire linked dataset, robust accuracy tests are performed, to assess the overall linkage quality.

4.2 Statistical accuracy of the linkage

The quality of the linkage was tested manually and using a receiver operating characteristic (ROC) curve by a team of statisticians working at CIDACS. The entire process is described below.

For the manual test, the team took a sample of 10,000 linked pairs (i.e. each pair represents a maternal death linked to a 100 Million Cohort record). Sampling was stratified by similarity scores, to enable assessment of the linkage quality for a range of similarity scores. The sampling process was developed in five stages:

Similarity scores generated in the linkage process were divided into five strata;

The number of observations in each was used to decide the stratum range;

- A variable was created for each linked record pair, to indicate the stratum of similarity to which the record pair belonged.
- The proportion of records in each similarity stratum was calculated.
- A random sample proportional to the size of the stratum was taken, within each similarity stratum.

In the random sample of 10,000 linked pairs, three of the five items of information used for the linkage (beneficiary's name, mother's name, and date of birth) were checked for each pair. Three new variables were created, indicating whether the information on the two databases (100 Million Cohort and SIM) agreed for each of the three variables separately, assigning a value for each variable of 1 for agreement, and 0 for disagreement. Records with agreement in all three variables were declared a true match and those with some disagreement were investigated further. For disagreements in the name fields, these were considered to agree when the names in the two databases contained different letters with similar phonetics. For foreign or uncommon names, disagreements of up to three digits were accepted (and the agreement variable was reset to 1, indicating agreement). For disagreements in the date of birth field, differences of only one number were accepted.

For remaining disagreements in the name fields, if any of the following occurred:

© 2023 Alves FJO et al. *JAMA Network Open*.

- The mother's name was completely different in the two databases.
- Three or more different letters in the name in the two databases.
- Completely different surnames in the two databases
- One of the pairs of records did not contain a surname in the two databases.
- The surname contained two or more abbreviations in one of the pairs of records, or different surnames.

The records were then declared to be a false match for remaining disagreements in the date of birth field, if any of the following occurred:

- At least one of the digits in the year was different, resulting in an age difference of at least 7 (seven) years; or
- At least one digit in the month was different, resulting in a difference of at least 10 (ten) months; or,
- Both the month and year were different.

The records were then declared to be a false match.

In addition to these cases, all other records with some disagreement were declared true matches following manual inspection. At this stage, if any doubts remained, they were reanalysed using two extra matching variables: sex and municipality.

ROC curve

Following the manual verification process, the record linkage algorithm sensitivity and specificity were estimated for a range of cut-off values (the criteria for declaring a true match using the record linkage algorithm), viewing the result of the manual verification as the gold standard classification of the links (eTable 18). Using these estimated specificities and sensitivities, ROC curves were constructed to identify the global accuracy (as measured by the area under the curve) of the results obtained by the similarity score (eFigure 3).

From the ROC curve, the optimal cut-off point of 0.92 (ROC curve area [Sensitivity/Specificity]: 0.923 [0.983/0.949]) was identified. Using this optimal cut-off to declare matches, 97.8% of the linked pairs were estimated to be true matches, and 2.2% were estimated to be false matches. An estimated 5% of the true matches were not linked (eFigure 3).

© 2023 Alves FJO et al. *JAMA Network Open*.

eTable 18. Accuracy Analysis of the Linkage Between *Cadastro Único* and the Mortality Information System in a Sample of 10 000 Record Pairs

**Cut-off point**	**Specificity; sensitivity**	**Total matches (%)**	**True matches (% of linked cases)**	**False matches (% of linked cases)**	**Lost matches (% of true matches)**
≥0.83	SP=0.459; S=0.996	7551 (75.5)	4686 (62.1)	2865 (37.9)	17 (0.4)
≥0.84	SP=0.576; S=0.993	6916 (69.2)	4668 (67.5)	2248 (32.5)	35 (0.7)
≥0.85	SP=0.692; S=0.990	6290 (62.9)	4657 (74.0)	1633 (26.0)	46 (1.1)
≥0.86	SP=0.789; S=0.987	5759 (57.6)	4641 (80.6)	1118 (19.4)	62 (1.3)
≥0.87	SP=0.863; S=0.977	5322 (53.2)	4595 (86.3)	727 (13.7)	108 (2.3)
≥0.88	SP=0.908; S=0.973	5060 (50.6)	4575 (90.4)	485 (9.6)	128 (2.7)
≥0.89	SP=0.941; S=0.969	4872 (48.7)	4557 (93.5)	315 (6.5)	146 (3.1)
≥0.90	SP=0.961; S=0.964	4741 (47.4)	4534 (95.6)	207 (4.4)	169 (3.6)
≥0.91	SP=0.974; S=0.956	4638 (46.4)	4498 (97.0)	140 (3.0)	205 (4.4)
≥0.92	SP=0.981; S=0.950	4570 (45.7)	4470 (97.8)	100 (2.2)	233 (5.0)
≥0.93	SP=0.986; S=0.940	4495 (45.0)	4423 (98.4)	72 (1.6)	280 (6.0)
≥0.94	SP=0.989; S=0.919	4381 (43.8)	4323 (98.7)	58 (1.3)	380 (8.0)
≥0.95	SP=0.991; S=0.895	4258 (42.6)	4211 (98.9)	47 (1.1)	492 (10.5)
≥0.96	SP=0.995; S=0.855	4049 (40.5)	4022 (99.3)	27 (0.7)	681 (14.5)
≥0.97	SP=0.998; S=0.750	3540 (35.4)	3527 (99.6)	13 (0.4)	1176 (25.0)
≥0.98	SP=0.998; S=0.603	2844 (28.4)	2835 (99.7)	9 (0.3)	1868 (39.7)

© 2023 Alves FJO et al. *JAMA Network Open*.

eFigure 3. ROC Curve of the Linkage Between Mortality and *Cadastro Único* Between 2001 and 2015

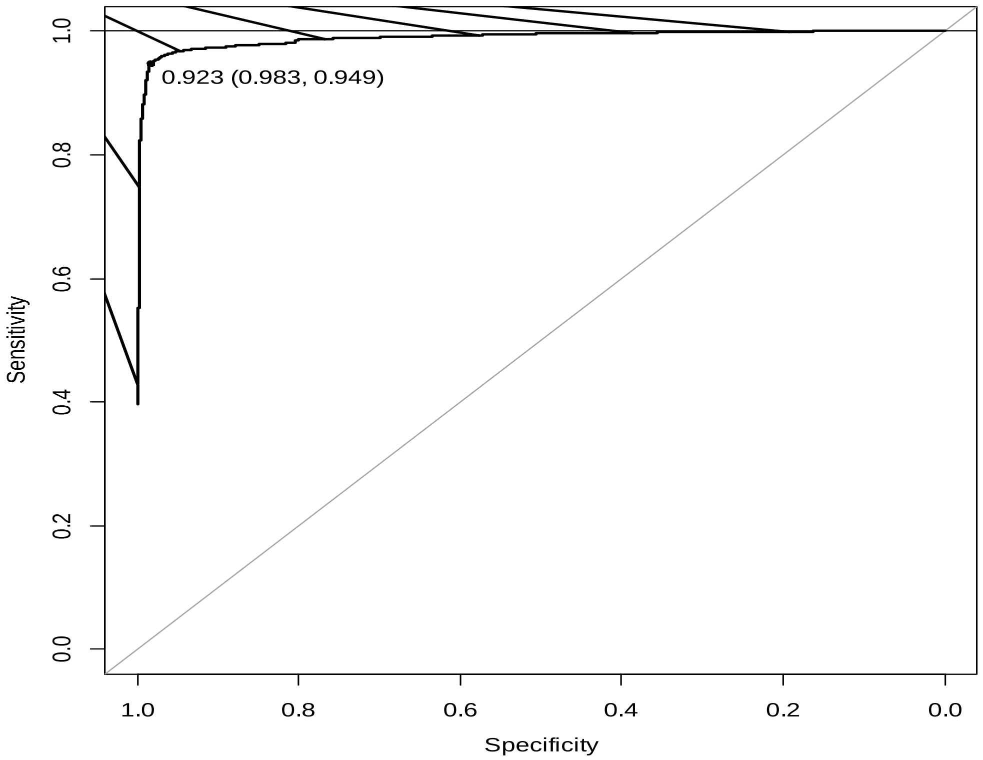

© 2023 Alves FJO et al. *JAMA Network Open*.

eReferences

1- Brazil. MDS. Bolsa Família [Internet]. Available at: http://www.mds.gov.br/bolsafamilia

2- Falcão IR, Ribeiro-Silva RC, Alves FJO, et al. Evaluating the effect of Bolsa Familia, Brazil's conditional cash transfer programme, on maternal and child health: A study protocol. *PloS one.* 2022;17(5):e0268500.

3- Ramos D, da Silva NB, Ichihara MY, et al. Conditional cash transfer program and child mortality: A cross-sectional analysis nested within the 100 Million Brazilian Cohort. *PLoS Med.* 2021;18(9):e1003509.Davide

4- Jesus GS, Pescarini JM, Silva AF, et al. The effect of primary health care on tuberculosis in a nationwide cohort of 7·3 million Brazilian people: a quasi-experimental study. *The Lancet Global Health.* 2022;10(3):e390-e397.

5- Ho, D. E. et al. Matching as nonparametric pre-processing for reducing model dependence in parametric causal inference. **Political analysis,** v. 15, n. 3, p. 199-236, 2007. ISSN 1047-1987.

6- Rosenbaum, P. R.; Rubin, D. B. Reducing Bias in Observational Studies Using Subclassification on the Propensity Score. **Journal of the American Statistical Association,** v. 79, n. 387, p. 516-524, 1984/09/01 1984. ISSN 0162-1459. Available at: < https://www.tandfonline.com/doi/abs/10.1080/01621459.1984.10478078 >.

7- Brookhart, M. Alan, et al. "Confounding control in healthcare database research: challenges and potential approaches." *Medical care* 48.6 0 (2010): S114.Stürmer, Til, et al. "Propensity scores for confounder adjustment when assessing the effects of medical interventions using nonexperimental study designs." *Journal of internal medicine* 275.6 (2014): 570-580.

8- Austin, Peter C. "An introduction to propensity score methods for reducing the effects of confounding in observational studies." *Multivariate behavioral research* 46.3 (2011): 399-424

9- .Lucene A. A high-performance, full-featured text search engine library. URL: http://lucene apache org 2005;

© 2023 Alves FJO et al. *JAMA Network Open*.
