# Supplementary material for: Association of Conditional Cash Transfers With Maternal Mortality Using the 100 Million Brazilian Cohort
Source: JAMA Netw Open. 2023 Feb 23;6(2):e230070. doi: 10.1001/jamanetworkopen.2023.0070 (PMC9951038; doi:10.1001/jamanetworkopen.2023.0070)
Supplement: Supplement 2. — Data Sharing Statement [file jamanetwopen-e230070-s002.pdf]

Data Sharing Statement

Alves. Association of Conditional Cash Transfers With Maternal Mortality Using the 100 Million Brazilian Cohort. *JAMA Netw Open*. Published February 23, 2023.
doi:10.1001/jamanetworkopen.2023.0070

Data
Data available: Yes
Data types: Data dictionary
How to access data: All data were obtained from Centro de Integracao de Dados e Conhecimentos para Saude (CIDACS). Restrictions apply to access to these data, which contains sensitive information, were licensed for exclusive use in the current study and, due to privacy regulations from the Brazilian Ethics Committee are not openly available. Upon reasonable request and with express permission from CIDACS (mail to cidacs.curadoria@fiocruz.br) and approval from an ethical committee, controlled access to the data is possible.
When available: With publication

Supporting Documents
Document types: Other (please specify)
Additional Information: All data were obtained from Centro de Integracao de Dados e Conhecimentos para Saude (CIDACS). Restrictions apply to access to these data, which contains sensitive information, were licensed for exclusive use in the current study and, due to privacy regulations from the Brazilian Ethics Committee are not openly available. Upon reasonable request and with express permission from CIDACS (mail to cidacs.curadoria@fiocruz.br) and approval from an ethical committee, controlled access to the data is possible.
How to access documents: All data were obtained from Centro de Integracao de Dados e Conhecimentos para Saude (CIDACS). Restrictions apply to access to these data, which contains sensitive information, were licensed for exclusive use in the current study and, due to privacy regulations from the Brazilian Ethics Committee are not openly available. Upon reasonable request and with express permission from CIDACS (mail to cidacs.curadoria@fiocruz.br) and approval from an ethical committee, controlled access to the data is possible.
When available: With publication

Additional Information
Who can access the data: All data were obtained from Centro de Integracao de Dados e Conhecimentos para Saude (CIDACS). Restrictions apply to access to these data, which contains sensitive information, were licensed for exclusive use in the current study and, due to privacy regulations from the Brazilian Ethics Committee are not openly available. Upon reasonable request and with express permission from CIDACS (mail to cidacs.curadoria@fiocruz.br) and approval from an ethical committee, controlled access to the data is possible.
Types of analyses: specified purpose
Mechanisms of data availability: after aproval of a proposal
Any additional restrictions: All data were obtained from Centro de Integracao de Dados e Conhecimentos para Saude (CIDACS). Restrictions apply to access to these data, which contains sensitive information, were licensed for exclusive use in the current study and, due to privacy regulations from the Brazilian Ethics Committee are not openly available. Upon reasonable request and with express permission from CIDACS (mail to cidacs.curadoria@fiocruz.br) and approval from an ethical committee, controlled access to the data is possible.
